# Case Report: Improved hearing in a rare, adult *IDH2*-mutant brainstem astrocytoma successfully treated with radiation and temozolomide

**DOI:** 10.3389/fonc.2025.1555986

**Published:** 2025-07-08

**Authors:** Takuya Okada, Manabu Natsumeda, Hidemoto Fujiwara, Nayuta Higa, Toshiaki Akahane, Yuki Watabe, Kaoru Tomikawa, Kyoka Nishita, Yoshihiro Tsukamoto, Shinsuke Ohshima, Arata Horii, Akihide Tanimoto, Ryosuke Hanaya, Hiroshi Shimizu, Akiyoshi Kakita, Makoto Oishi

**Affiliations:** ^1^ Department of Neurosurgery, Brain Research Institute, Niigata University, Niigata, Japan; ^2^ Advanced Treatment of Neurological Diseases Branch, Brain Research Institute, Niigata University, Niigata, Japan; ^3^ Department of Neurosurgery, Kagoshima University Graduate School of Medical and Dental Sciences, Kagoshima, Japan; ^4^ Department of Pathology, Kagoshima University Graduate School of Medical and Dental Sciences, Kagoshima, Japan; ^5^ Department of Otolaryngology, Head and Neck Surgery, Niigata University Graduate School of Medical and Dental Sciences, Niigata, Japan; ^6^ Department of Pathology, Brain Research Institute, Niigata University, Niigata, Japan

**Keywords:** adult diffuse intrinsic pontine glioma, non-canonical IDH mutation, 2-hydroxyglutarate, magnetic resonance spectroscopy, temozolomide sensitivity

## Abstract

**Introduction:**

Brain stem gliomas harboring IDH mutations can be sensitive to temozolomide (TMZ) treatment, unlike their H3K27-altered counterparts, so distinguishing the two is essential.

**Case presentation:**

Here, we report an adult brainstem glioma patient whose hearing loss normalized after treatment. He presented with gradual left hearing loss from two years before, and magnetic resonance (MR) images showed a diffuse mass lesion involving the pons to left middle cerebral peduncle, including the vestibular and cochlear nuclei. On MR spectroscopy (MRS), 2-hydroxyglutarate (2HG) was elevated to 3.602 mM, suggesting an IDH-mutant glioma. Subsequently, an open biopsy was performed via the lateral suboccipital approach, and the pathological diagnosis was astrocytoma, IDH-mutant, CNS WHO grade 3. Molecular analysis revealed a non-canonical *IDH2* R172S mutation. Left hearing improved from 87.5 dB to 8.3dB by 6-frequency pure tone audiogram (PTA) and 90% speech discrimination at 35 dB after concomitant TMZ and radiation treatment, followed by 12 cycles of adjuvant TMZ treatment. 2HG also decreased to 0.186 mM on MRS after treatment determining treatment strategy.

**Discussion:**

Studies have shown that as high as 31% of adult brainstem gliomas are IDH mutant, with most of these mutations being non-canonical *IDH1/2* mutations. Approximately 70% of IDH-mutant astrocytomas are known to harbor a methylated O6-methylguanine-DNA-methyltransferase (*MGMT*) promoter and respond to TMZ treatment, whereas almost all H3K27M-mutant diffuse midline gliomas have unmethylated *MGMT* promoters and generally are not sensitive to TMZ treatment. Detection of 2HG by MRS and molecular analysis, including non-canonical *IDH1/2* mutations, were helpful in determining treatment response in this adult brainstem glioma case. Notably, hearing loss normalized after TMZ treatment.

**Conclusion:**

The diagnosis of IDH-mutant brainstem gliomas by MRS and integrated analysis of surgically obtained specimens is essential to determine the proper treatment of these rare cases.

## Introduction

Adult brainstem gliomas are rare, accounting for less than 2% of all adult gliomas (1). Large-scale studies reporting the genetic profile of adult brainstem gliomas are scarce (2). Their pediatric counterparts, which are known to harbor recurrent alterations of *H3F3A* K27M (3–5), are now classified along with diffuse gliomas arising from the thalamus (6) and spine (7) as diffuse midline gliomas, H3K27-altered (8). These gliomas almost universally have unmethylated O6-methylguanine-DNA-methyltransferase (*MGMT*) promoter (9, 10) and generally do not respond to the oral alkylating agent temozolomide (TMZ) (11, 12), with a dismal prognosis (13–17). A couple of studies have shown that mutations in *IDH1* and *IDH2* genes can be found in 18-31% of adult brainstem gliomas (18–20) and that a majority of these mutations are non-canonical *IDH1/2*-mutations (21, 22), compared to a high frequency of *ID*H1 R132H mutations in IDH-mutant supratentorial diffuse gliomas (23, 24). Determining the molecular profile of adult brainstem gliomas is very important clinically because IDH-mutant diffuse gliomas frequently harbor a methylated *MGMT* promoter and can be expected to respond to TMZ treatment (11, 25).

Here, we present a case of adult brainstem glioma with non-canonical *IDH2* mutation and treatment response to radiation and TMZ. Pre-operatively, 2-hydroxyglutarate (2HG) was detected by magnetic resonance spectroscopy (MRS). This rare case report provides rationale that biopsy and genetic testing should be performed in adult brainstem gliomas when feasible to obtain valuable information about treatment response.

## Case presentation

A 33-year-old Japanese male with no significant past medical history was referred to Niigata University Medical and Dental Hospital with progressive left hearing loss and tinnitus, which had been worsening for two years. A 6-frequency pure tone audiogram (PTA) was 87.5 dB in the left ear compared to 7.5 dB in the right, and only wave I could be identified on the left auditory brainstem response (ABR) (**Supplementary Figure 1A**).

**Figure 1:**
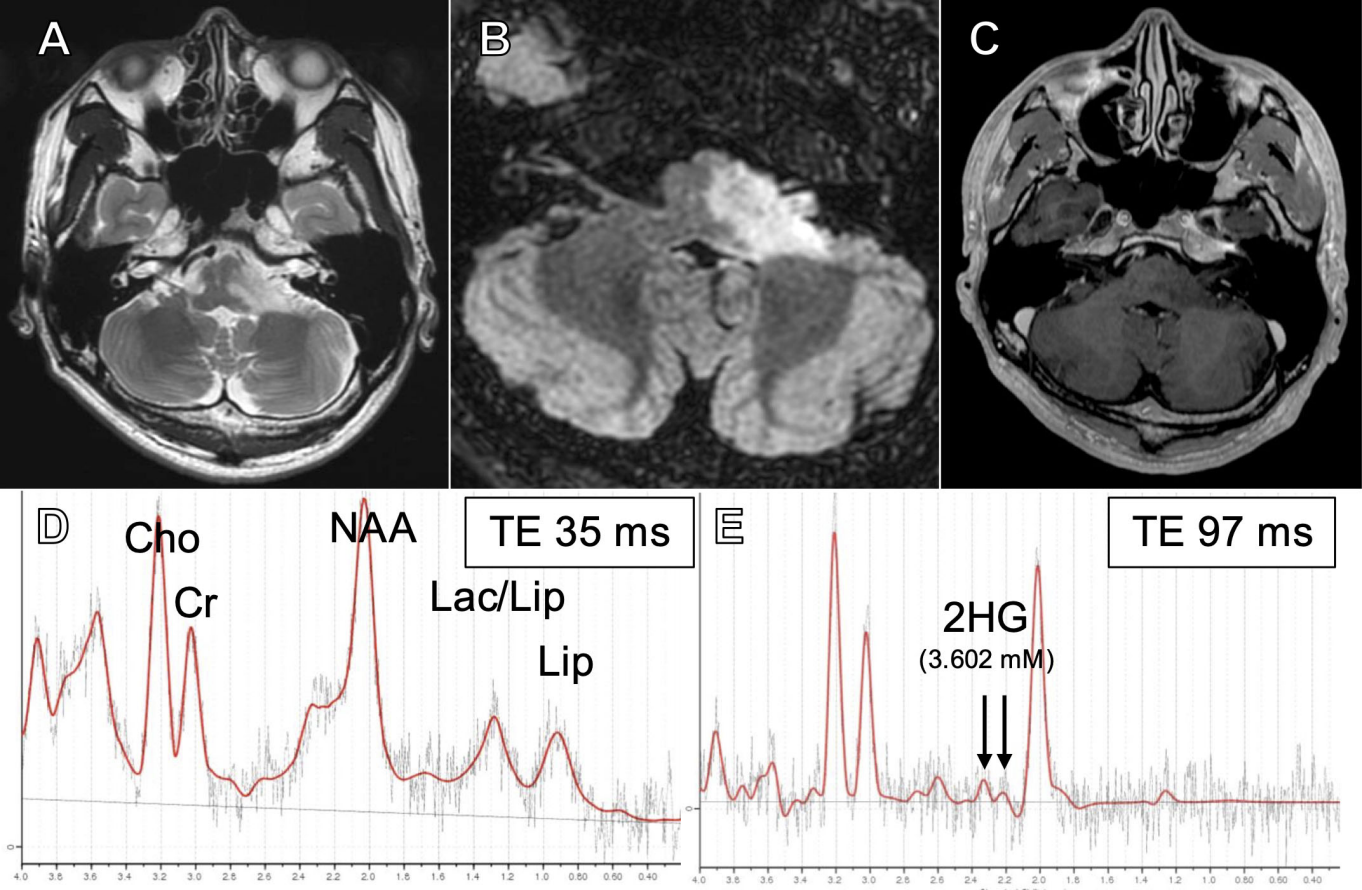
T2-weighted **(A)**, fluid-attenuated inversion recovery (FLAIR) **(B)**, and post-contrast **(C)** magnetic resonance (MR) images at presentation. Short TE (35 msec) single voxel MR spectroscopy (MRS) showing elevated Cho/Cr and decreased NAA **(D)** and intermediate TE (97 msec) SVMRS showing elevated 2-hydroxyglutarate **(E)**.

Magnetic resonance (MR) images showed a hyperintense lesion extending from the pons to the left middle cerebellar peduncle on T2-weighted and fluid attenuation inverted recovery (FLAIR) images, including the vestibular and cochlear nuclei at the rhomboid fossa (**Figures 1A**). Post-contrast images showed no enhancement (**Figure 1**). Magnetic resonance spectroscopy (MRS) showed an increased choline (Cho)-to-creatine (Cr) ratio and decreased N-acetyl aspartate (NAA), suggestive of a malignant brain tumor (**Figure 1**). In addition, on single voxel MRS with a TE at 97 msec to optimize the detection of 2HG (26), 2HG was detected (3.602 mM at TE =97 msec; S/N =7, Cramer-Rao lower bounds [CRLB] = 32%), suggesting the presence of IDH mutation (**Figure 1**). 2HG was also detected at a TE of 35 msec (5.692 mM; S/N = 6, CRLB = 42%), although the CRLB was high, suggesting the possible contamination of macromolecules.

A typical DIPG displays more than 50% T2/FLAIR hyperintensity of the ventral pons on MR images (27). In the present case, the tumor progressed to the left middle cerebellar peduncle. Furthermore, considering the results of the MRS and the relatively slow progression of symptoms, the clinicoradiographical presentation was atypical for H3K27-altered DMG of the brainstem. Therefore, we decided that a biopsy was necessary.

**Figure 2:**
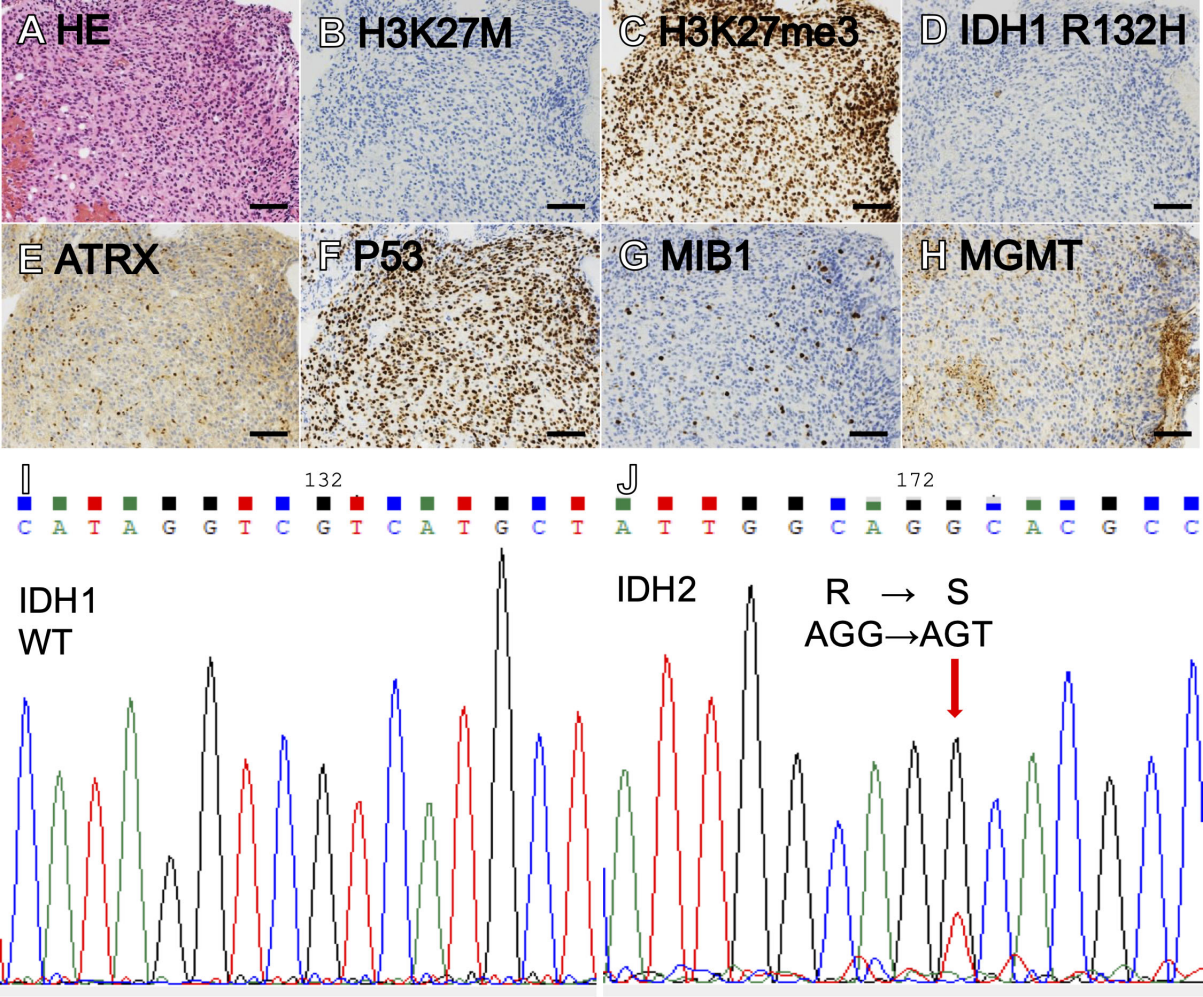
Hematoxylin-eosin (HE) staining showed a diffuse, astrocytic tumor with nuclear atypia and moderately increased cellularity **(A)**. The tumor showed negative H3K27M **(B)**, intact H3K27me3 **(C)**, negative IDH1 R132H **(D)**, loss of ATRX **(E)**, and marked staining for P53 **(F)**. MIB labeling index was 8% **(G)** and MGMT was positive in 5% of tumor cells **(H)**. Sanger sequencing showing *IDH1* wildtype **(I)** and *IDH2* R172S **(J)**. (Scale bars = 100 µm).

We performed a biopsy via the left lateral suboccipital approach, and small pieces of edematous tumor between the root exit zones of the V and VII/VIII complex were sampled. Hematoxylin and eosin (HE) sections showed diffuse astrocytic tumor with nuclear atypia (**Figure 2**). Immunohistochemically, the tumor cells were negative for H3K27M and positive for H3K27me3 (**Figures 2B**). Also, IDH1 R132H was negative, but ATRX staining was diminished and P53 was strongly positive, suggestive of an IDH-mutant astrocytoma (**Figures 2D**). The MIB-1 labeling index was 8% (**Figure 2**). MGMT was positive in only 5% of tumor cells (**Figure 2**), below the cutoff of 30% (28), suggesting that the *MGMT* promoter is methylated. Sanger sequencing (IRB approval #G2022-0012) revealed *IDH1* R132 wildtype and *IDH2* R172S mutation (**Figures 2I**). A bimodal DNA and RNA next-generation sequencing panel for integrative diagnosis of glioma (7, 29) was performed (IRB approval #C2023-0039), and *IDH2* R172S mutation (variant allele frequency [VAF] 48.1%), as well as *TP53* C275F (VAF 91.2%) and *ATRX* c.3809 + 1G>C (VAF 92.1%), were detected. There was no loss of *CDKN2A/B*. The integrated diagnosis was astrocytoma, IDH-mutant, CNS WHO grade 3.

**Figure 3:**
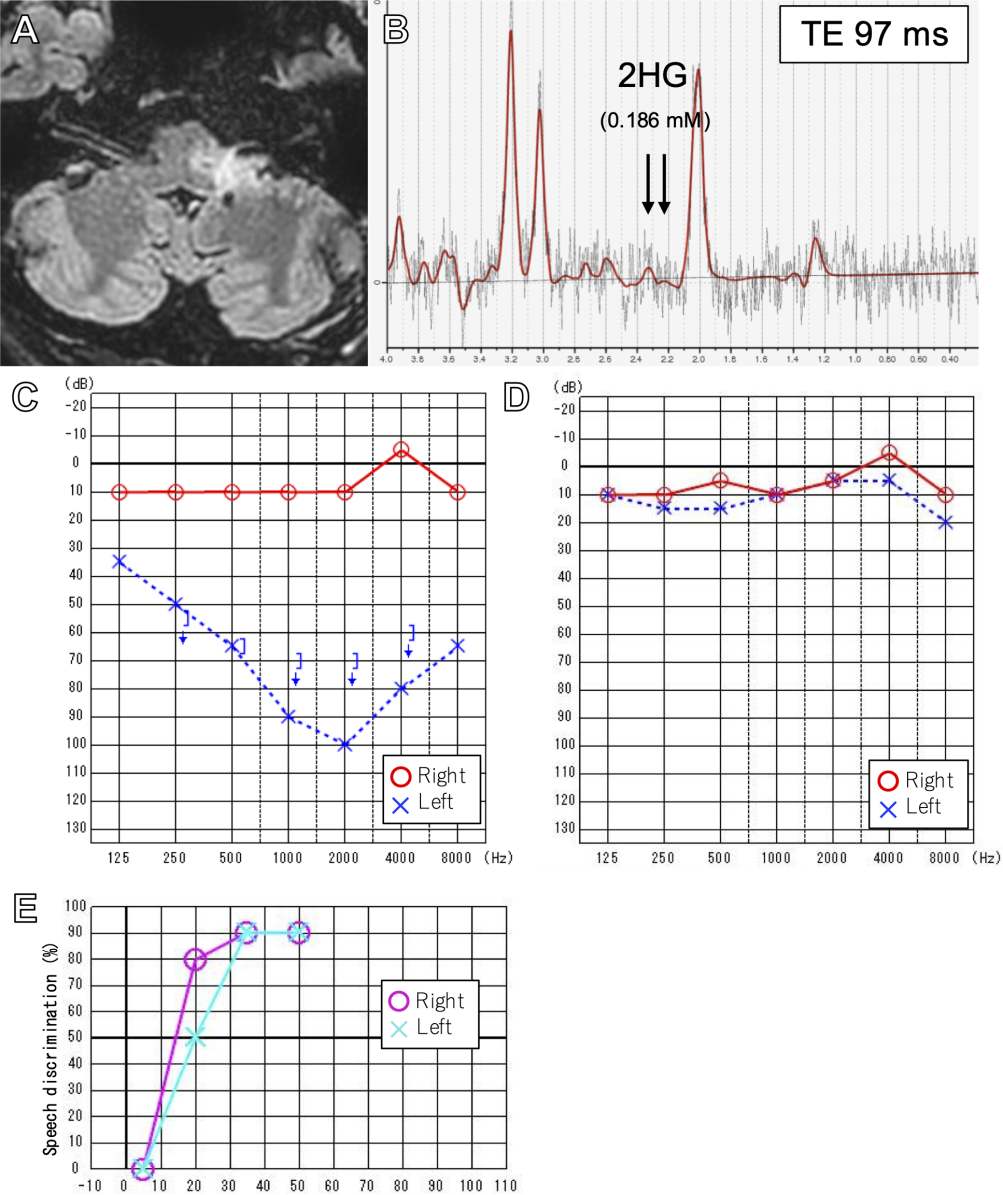
FLAIR image after 12 cycles of adjuvant temozolomide **(A)**. Intermediate TE (97msec) SVMRS after treatment showing decreased 2HG **(B)**. Pure tone audiometry before **(C)** and after treatment **(D)** showing normalization of left hearing. Speech discrimination after treatment was also normal **(E)**.

After the surgery, the patient experienced minimal hypesthesia of the left perioral area. The patient underwent concomitant TMZ and 54 Gy (30 fractions) of intensity-modulated radiation therapy (IMRT), followed by 12 courses of maintenance TMZ. Three months after the completion of treatment, the area of T2/FLAIR hyperintensity on MR images decreased by 51% by volumetric analysis (30) (**Figure 3**), and 2HG on MRS had drastically decreased to 0.186 mM, suggesting a treatment effect (**Figure 3**). After finishing TMZ treatment, the patient noticed a partial improvement in his left hearing. His left hearing loss had normalized from 87.5 dB on 6-frequency PTA (**Figures 3**) to 8.3 dB (**Figure 3**) and 90% speech discrimination at 35 dB (**Figure 3**). However, ABR findings remained unchanged, with only wave I identified on the left ABR (**Supplementary Figure 1**). The patient’s partial improvement of left hearing has persisted, and he is recurrence-free eight months after completion of treatment and 22 months from diagnosis.

## Discussion

In the present case, a dramatic response to radiation and temozolomide treatment and subsequent improved hearing were observed in a rare *IDH2* R172S-mutant brainstem glioma patient who presented with left hearing loss. Detection of 2HG by MRS served as a less-invasive adjunct to screen for potential IDH mutation, which was confirmed by integrated diagnosis of surgically obtained tissue.

A multicenter review by Shumacher et al. deemed that biopsies are rarely needed to diagnose pediatric brainstem lesions, and MR imaging would suffice in most cases (31). With the introduction of molecular analysis to brain tumor diagnosis, stereotactic and robot-assisted techniques have been implicated in the biopsy of brainstem lesions, however, with the potential for significant morbidity (32).

Up to 90% of pediatric DIPGs are known to harbor *H3F3A* K27M or *HIST1H3B* K27M mutations (3–5, 33). Almost all of these H3K27-altered DMGs have unmethylated *MGMT* promoters (9, 10) and are resistant to TMZ treatment (11, 12, 34). However, a significant percentage of IDH-mutant astrocytomas have been implicated in adult brainstem gliomas, with rates ranging from 18-31% in relatively large series (18–20). It is important to consider that most IDH-mutant astrocytomas arise after the second decade of life (35), likely contributing to the scarcity of IDH-mutant pediatric brainstem gliomas. Almost 70% of IDH-mutant astrocytomas are known to harbor methylated *MGMT* promoters (23), making them more likely to respond to TMZ treatment (11). In the present case, the diagnosis of IDH mutant astrocytoma was vital in determining treatment with TMZ.

Remarkably, hearing loss dramatically improved after radiation and TMZ treatment. This is especially surprising as hearing loss had been noted for over two years before the presentation. To date, dramatic improvement of cranial nerve signs after treatment has not been extensively reported in gliomas. Visual acuity markedly improved after bevacizumab treatment in 4 cases of pediatric optic pathway gliomas previously treated with chemotherapy or proton-beam radiation (36). Objective hearing improvement was observed in 8 out of 13 (61%) patients with hearing loss in neurofibromatosis type 2-related vestibular schwannomas after receiving bevacizumab (37). We speculate that in the present case, a reduction in compression of the auditory tract by the tumor due to treatment effects led to improved hearing. As seen in both pre- and post-treatment ABR, wave I, which originates from the cochlea, is preserved, suggesting that the cochlear periphery was not affected by the tumor. Different from insults to cochlear peripheries, central auditory pathways may have the potential to recover from injuries via mechanisms such as neural redundancy. Therefore, normal cochlea and reduction of auditory tract compression due to tumor shrinkage could bring about the improvement of hearing on PTA. However, the patient continues to subjectively claim that his left hearing is worse than the right, suggesting that the patient did not attain usable conversational hearing even after the TMZ treatment. Although the post-treatment speech discrimination test seems to be good even in the affected ear, it may be due to the usage of relatively simple syllables in the speech discrimination test in Japan.

In the present case, MRS was performed at presentation, and a high Cho/Cr ratio, decreased NAA, and accumulation of 2HG were noted, suggesting a malignant tumor with possible IDH mutation. We (38, 39) and others (26, 40–42) have succeeded in the detection of 2HG by MRS and also have reported the usefulness of 2HG detection by MRS in non-canonical IDH-mutant gliomas (41, 43). A report by Iwahashi et al. nicely illustrates the diagnosis of non-canonical IDH mutations in brainstem gliomas by MRS (44). 2HG detection by MRS is an important adjunct in diagnosing IDH-mutant brainstem astrocytomas because 72% of infratentorial IDH-mutant gliomas (21) and 59% of IDH-mutant brainstem gliomas (22) have been reported to harbor non-canonical IDH mutations. The *IDH2* R172S mutation found in the present case is extremely rare in gliomas. This mutation was not reported in a large series of 170 IDH-mutant gliomas from the US (24) and 286 from Japan (23). Banan et al. report 2 out of 42 (5%) infratentorial and 0 out of 50 (0%) supratentorial IDH-mutant gliomas to be *IDH2* R172S-mutant (21).

In addition to the less-invasive detection of 2HG by MRS, surgical biopsy of the lesion and integrated diagnosis of the specimen is strongly recommended when feasible. We have previously reported a specificity of 72.2-81.3% of 2HG accumulation by MRS to detect IDH mutation in gliomas (38, 43). Therefore, false-positive cases can exist. Also, the CRLB of 2HG detection in the present case was 32%, higher than the optimal <20% (26), further necessitating histological confirmation. In the present case, an open biopsy was safely performed, enabling pathological and molecular confirmation.

## Conclusion

Marked improvement of hearing was observed after TMZ and radiation treatment in a rare *IDH2* R172S-mutant, adult brainstem glioma case. Detection of 2HG by MRS is important for the less-invasive screening of IDH mutation, but a surgical biopsy is strongly recommended when feasible to determine the proper treatment.

## Data Availability

The raw data supporting the conclusions of this article will be made available by the authors, without undue reservation.

## Supplementary material

Supplementary Figure 1 Auditory brainstem response before **(A)** and after **(B)** treatment.
