# Supplementary figures and images for: Case Report: Improved hearing in a rare, adult IDH2-mutant brainstem astrocytoma successfully treated with radiation and temozolomide
Source: Front Oncol. 2025 Jul 8;15:1555986. doi: 10.3389/fonc.2025.1555986 (PMC12279812; doi:10.3389/fonc.2025.1555986)

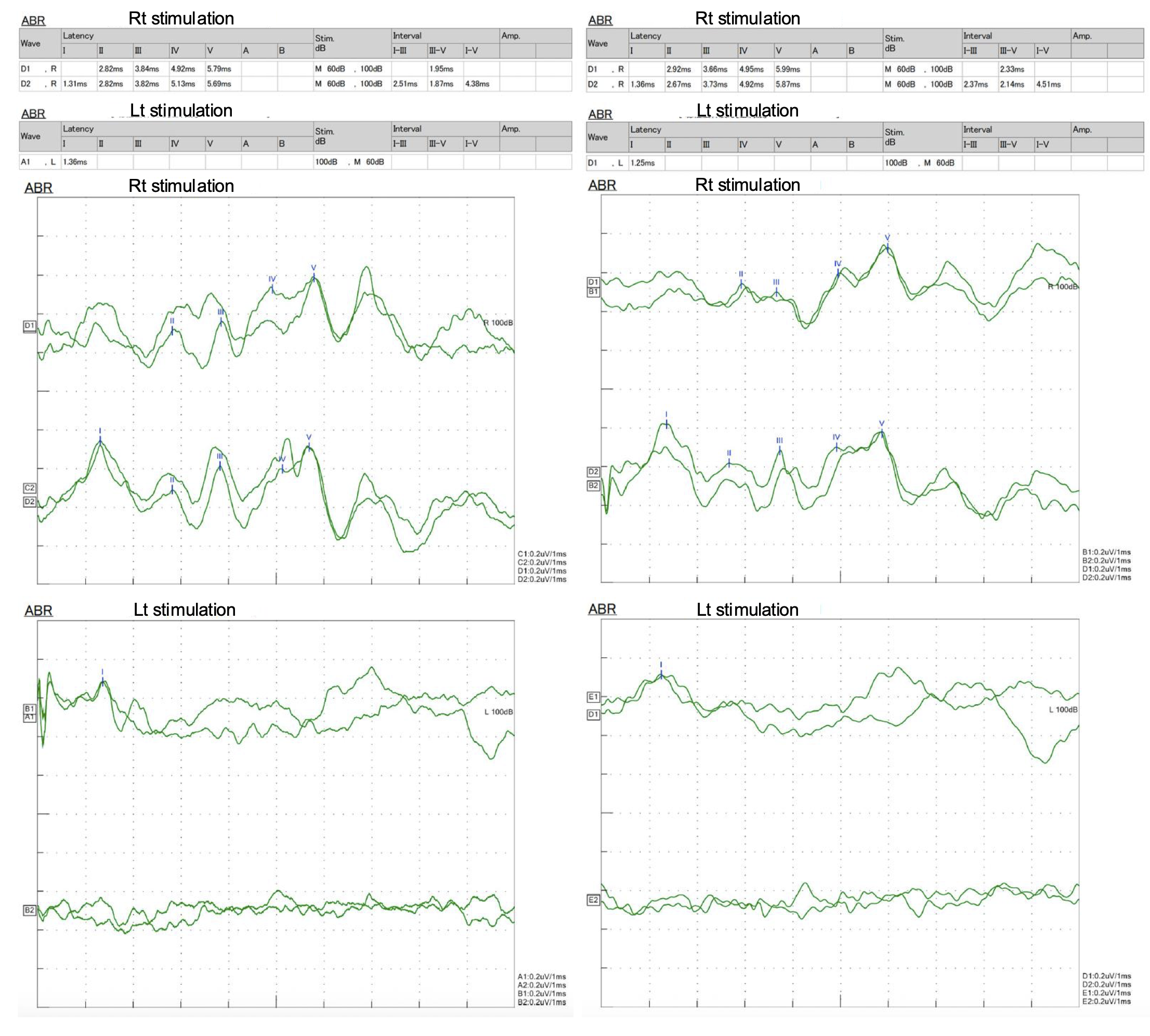

Supplement: Supplementary Figure 1 — Auditory brainstem response before (A) and after (B) treatment. [file Image1.tif]
